# Validation of a mathematical model for understanding intracranial pressure curve morphology

**DOI:** 10.1007/s10877-019-00342-8

**Published:** 2019-07-01

**Authors:** Mårten Unnerbäck, Johnny T. Ottesen, Peter Reinstrup

**Affiliations:** 1 Department of Clinical Sciences Lund, Intensive Care and Perioperative Medicine, Lund University, Skåne University Hospital, IPV SUS Malmö, Inga Marie Nilssons gata 47, 205 02 Malmö, Sweden; 2 Department of Science and Environment, Roskilde University, Roskilde, Denmark; 3 Department of Clinical Sciences Lund, Department of Neurosurgery, Lund University, Skåne University Hospital, Lund, Sweden

**Keywords:** Cerebral blood flow, Phase contrast magnetic resonance imaging, Intracranial pressure, Mathematical modelling

## Abstract

The physiology underlying the intracranial pressure (ICP) curve morphology is not fully understood. Recent research has suggested that the morphology could be dependent on arterial cerebral inflow and the physiological and pathophysiological properties of the intracranial cavity. If understood, the ICP curve could provide information about the patient’s cerebrovascular state important in individualizing treatment in neuro intensive care patients. A mathematical model based on known physiological properties of the intracranial compartment was created. Clinical measurements from ten neuro intensive care patients in whom intracranial arterial blood inflow, venous blood outflow and cerebrospinal fluid flow over the foramen magnum had been measured with phase contrast MRI, concomitant with ICP measurements were used to validate the model. In nine patients the mathematical model was able to create an ICP curve mimicking the measured by using arterial intracranial inflow and adjusting physiological parameters of the model. The venous outflow and cerebrospinal fluid (CSF) flow over the foramen magnum predicted by the model were within physiologically reasonable limits and in most cases followed the MRI measured values in close adjunct. The presented model could produce an ICP curve in close resemblance of the in vivo measured curves. This strengthens the hypothesis that the ICP curve is shaped by the arterial intracranial inflow and the physiological properties of the intracranial cavity.

## Introduction

The first cannulation of the ventricular system in order to measure intracranial pressure (ICP) was described by Guillume and Janny in 1951 [1]. Lundberg developed the method further and since then monitoring of the intracranial pressure invasively remains one of the corner stones of neuro intensive care [2]. Guidelines recommend keeping the cerebral perfusion pressure (CPP) at a level believed to avoid an insufficient cerebral blood flow [3]. This may be achieved by either manipulating the ICP, the arterial blood pressure (ABP) or a combination of both. It is reasonable to believe that the CPP level required to avoid a dangerously low cerebral blood flow (CBF) varies between individuals, depending on pre-existing medical conditions, age and the pathophysiological state at the time being. Defining the auto regulatory state of the patient has been introduced and used to guide therapy [4].

The morphology of the ICP curve has been explored in several studies, yet the physiological causes behind its features still remain unclear [5]. Measured over the cardiac cycle the ICP curve usually displays several distinct peaks. Early studies suggested a correlation between these peaks and heart valves opening and closing [6]. Transferring of the ABP to the intracranial compartment was early considered to cause the rise in the ICP curve over the cardiac cycle, with different damping characteristics for the frequencies within the ABP curve explaining the multiple peaks [7, 8]. Other researchers have focused on the change in intracranial volume over the cardiac cycle as a cause for the ICP curve, and in a recent study a correlation between rapid changes in intracranial volume and the initial ICP peak was found [9, 10].

If the physiology behind the ICP curve is fully understood it could be possible to enhance the understanding of the individual’s cerebrovascular physiological state, thereby making it possible to individualize treatment. In one study a correlation between low CBF and distinct features of the ICP curve was found and in another recently published study a correlation between the pulsatile component of CBF and the ICP curve morphology could be demonstrated [11, 12].

Research in this area is however difficult, both in practical and ethical aspects. Cine phase contrast MRI makes it possible to measure flow in vessels [13]. This technique was then used to measure CBF by measuring the flow in the internal carotid as well as the vertebral arteries or the basilar artery, which together supply the brain with blood [14]. Cine phase contrast MRI has been used in several studies to measure arterial inflow and venous outflow as well as cerebrospinal fluid (CSF) flow over the foramen magnum [15–17]. The technology offers the opportunity to measure the flows over the cardiac cycle and thereby calculate intracranial volume changes [9].

These phase contrast measurements are sometimes done in neuro intensive care patients who are monitored invasively regarding ICP. Clinical data from these individuals may be used for retrospective analysis but provides only small samples of observational data [10, 12]. Animal models could be considered but are cumbersome and the application of data in humans questionable.

Mathematical modelling is a method which has the benefit of creating an environment where experimental studies can be conducted without using animal models or jeopardizing human safety. This methodology has been used to explore different physiological properties of the intracranial system [18–21]. By creating a mathematical model of the intracranial cavity, it is possible to change different variables such as arterial inflow and capacitance, compliance and resistance on both the arterial and venous side of the cerebral vascular system to evaluate in what way these affect the ICP curve morphology. Indeed, the method has been used to analyze changes in ICP over longer periods of time [22–24], but not over the short time span of one cardiac cycle. Since all mathematical models in some sense represent a simplification of a complex reality the model has to be validated. If the model can be shown to reasonably resemble the reality, it could then be used in further research.

In this study we created a new mathematical model, mimicking known physiological features regarding the different fluid compartments within the intracranial space and compared this to data acquired from neuro intensive care patients.

## Methods

Ethical permission to extract data from the clinical database at Skåne University Hospital and analyse it was sought and granted. (2014/403).

### Mathematical modelling

**Fig. 1:**
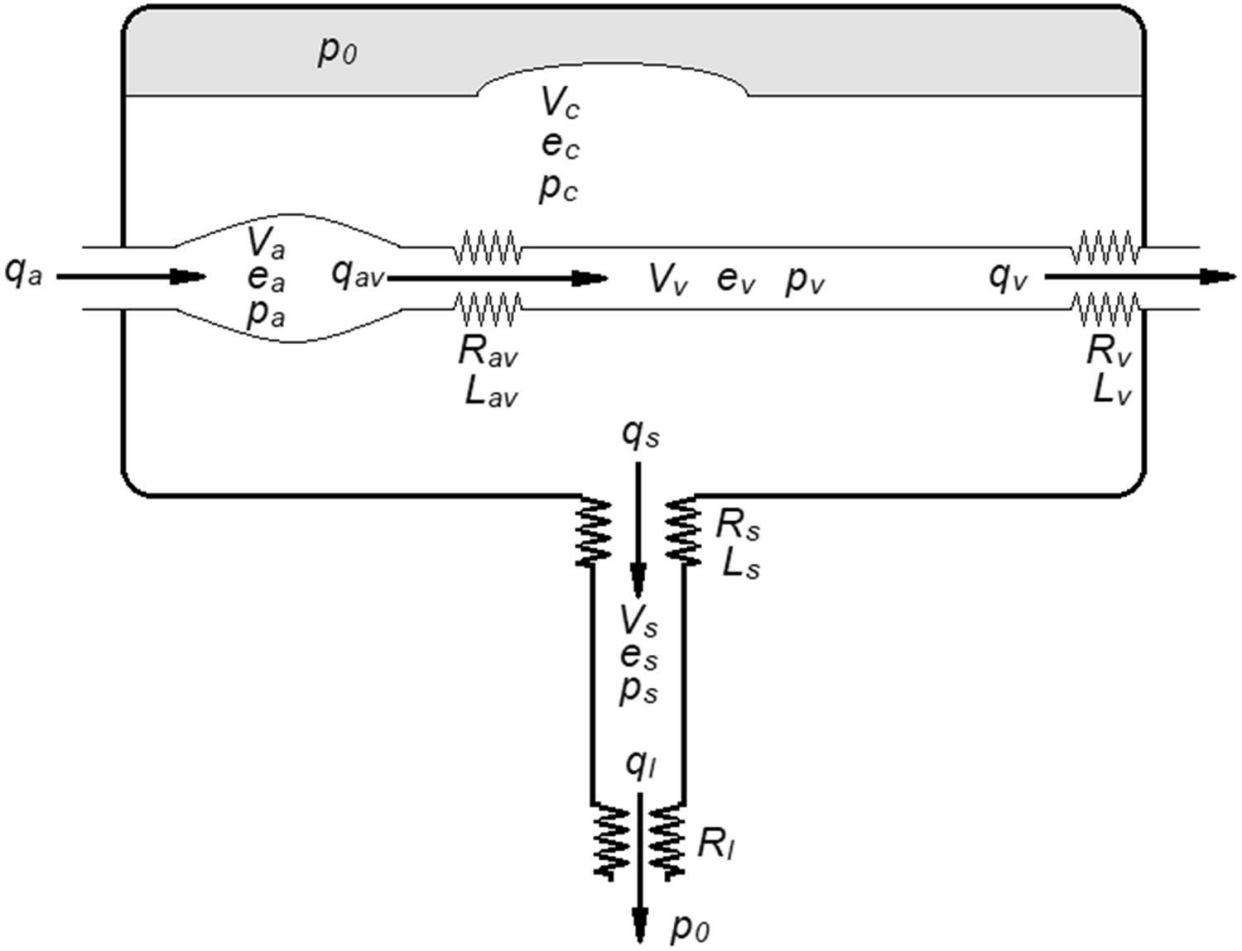
A drawing of the mathematical model. The different compartments illustrated in a drawing. Flow (q), volume (v), elastance (e), pressure (p), resistance (R) and impedance (L) are all denoted by the respective compartment or flow direction. P_0_ equals the pressure in the brain tissue and outside the spinal cord compartment. Note that the actual flow q_s_ is bidirectional (as all flow may be) and the direction depends on the state of the model. However, the arrows indicate the convention of positive flow for calculating purposes

A mathematical model describing the physiological features of the different fluid compartments within the cranial cavity was created. The model is thoroughly described in the appendix. The input variable is cerebral arterial inflow. This inflow causes a rapid expansion of intracranial arterial blood volume due to the flow resistance from the arteries to the veins. Since the cranial compartment could be considered as closed and the content virtually incompressible, this inflow must be compensated by an increased venous outflow, as well as an outflow of cerebrospinal fluid at the foramen magnum level. The inflow initially supersedes the outflow and causes an increase in cerebral blood volume (CBV) and ICP and is therefore also compensated by another compensatory mechanism, such as a slight movement of the brain caudally [25]. Since the compensatory mechanisms all are dependent on the arterial inflow, there exists a small delay in these mechanisms, introducing inertia in the model. This delay in response has been reported in previous studies [10, 26]. A representation of the model is presented in Fig. 1.

The CSF flow through the foramen magnum is bidirectional, as the main part returns during diastole. Since the resistance from a large container into a smaller is higher than the resistance from a small to a larger container, the resistance through the foramen magnum was set higher when CSF flow out from the cranial cavity [27]. Similarly, the impedance may be different whether flow is out of a small compartment into a larger one or vice versa. [28] The resistance to venous outflow is in the model indirectly dependent on the difference in venous pressure and ICP, since a larger difference likely due to a higher ICP will compress the veins, thus decreasing the cross-sectional area and thereby increase the venous resistance. [28–32].

The model was created based on these physiological principles as a software using Mat lab R2015b. A standard Pc computer was used to run the software.

To test the model, it was compared to data acquired through clinical measurements. The database at the neuro intensive care unit in Skåne University Hospital containing all patients who has been examined with cine phase contrast MRI regarding CBF was searched. Those patients who had adequate MRI examinations regarding both cerebral arterial blood flow, cerebral venous blood flow and CSF flow over the foramen magnum with simultaneous invasive ICP measurements were included.

### MRI measurements

All patients had been examined with a Philips Intera 1.5T MRT using phase contrast. A slice placed perpendicular to the internal carotid and vertebral arteries with a 256 × 128 matrix was used. Velocity encoding value had been set to 90 cm/s for blood flow measurements and 8 cm/s for CSF flow measurements. A flip angel of 15° and a TR of 26 ms had been used. Each examination took approximately 2 min. Arterial and venous blood flow had been measured immediately before CSF flow measurement during what was considered a physiological stable time period.

The MRI examinations were analysed using SEGMENT v 2.0 R5432, a freely available software [33]. One examiner performed the MRI examinations blinded to the ICP curves. The internal carotid arteries, vertebral arteries, internal jugular veins and the cerebrospinal canal were identified and region of interests surrounding these structures were placed manually on the phase contrast images. The flow was then acquired using the software, pixel by pixel with a temporal resolution of 30–35 time points per individual.

To calculate the arterial inflow, the total flow in the internal carotid and vertebral arteries was summarized. The venous outflow was calculated by summarizing the flow in the internal jugular veins. Since the venous outflow from the cranial cavity may take different paths the total venous outflow was multiplied by a factor to equal the total arterial inflow over the cardiac cycle [9, 17, 34].

### ICP measurements

All included patients had their ICP measured with a 8-F tunnelled intraventricular catheter (HanniKath, Smiths Medical Deutschland GmbH) inserted through a cranial burr hole. The catheter was in all cases connected to a CSF drainage set with a micro transducer (HanniSet, Smiths Medical Deutschland GmbH). The uppermost point of the cranium was used as zeroing point against atmospheric pressure. The signal from the ICP pressure transducer was taken out from the MR-investigation room through a radiofrequency filter at the penetration panel in the radiofrequency shielding enclosure into the MR-control room. The signal was then digitally registered using a Philips Intellivue MP70 STAD with a sampling rate of 125 Hz and immediately stored in a database. The ICP curves registered during the MRI examinations were manually identified and extracted. All ICP curves registered over one respiratory cycle during the MRI examination were summarized and a mean ICP curve was calculated.

Data from the included patients have previously been published in an observational study [12].

### Mathematical modelling

Cerebral arterial inflow calculated from the MRI examinations were feed into the mathematical model as an invariable input. All other variables are output from the model. A subset of the model parameters, describing physiological characteristics of the system, were varied from patient to patient. The model was then repeated after every adjustment until a steady state was achieved, which took up to 120 cardiac cycles. The measured and simulated ICP curves were compared by using R^2^ values. Automatic optimization routines were investigated but all were sensitive to a suitable a priori estimate for the parameters (a common problem in parameter estimation for nonlinear differential equations involving many parameters). First, we made some rough parameter estimation based on physiologically reasons and qualified guesses. Then we performed an investigation of parameter sensitivities around this set of a priori estimated parameters showing that 5 of these parameters were insensitive leaving 22 for individual posterior parameter estimation. Since this is still a large number of parameters automatic routines didn’t work properly, thus we refitted the remaining parameters by hand to achieve a fair a priori estimate and refine that by the Shuffle Complex Evolution method. For half of the patients, less than five parameters needed to be adjusted and in average between six and seven parameters were varied. This process was repeated until an ICP curve resembling the measured ICP curve as much as possible was achieved and output data regarding CSF flow and venous outflow was subsequently sampled at this point. The output data was then compared to measured values.

### Statistical analysis

For statistical analysis IBM SPSS Statistics for Windows, Version 22.0. (IBM Corp, USA) was used. Linear regression was used to test correlation. The Student´s *t* test was used to test the null hypothesis between samples. All values are given as mean with standard deviation, unless otherwise stated.

## Results

**Fig. 2:**
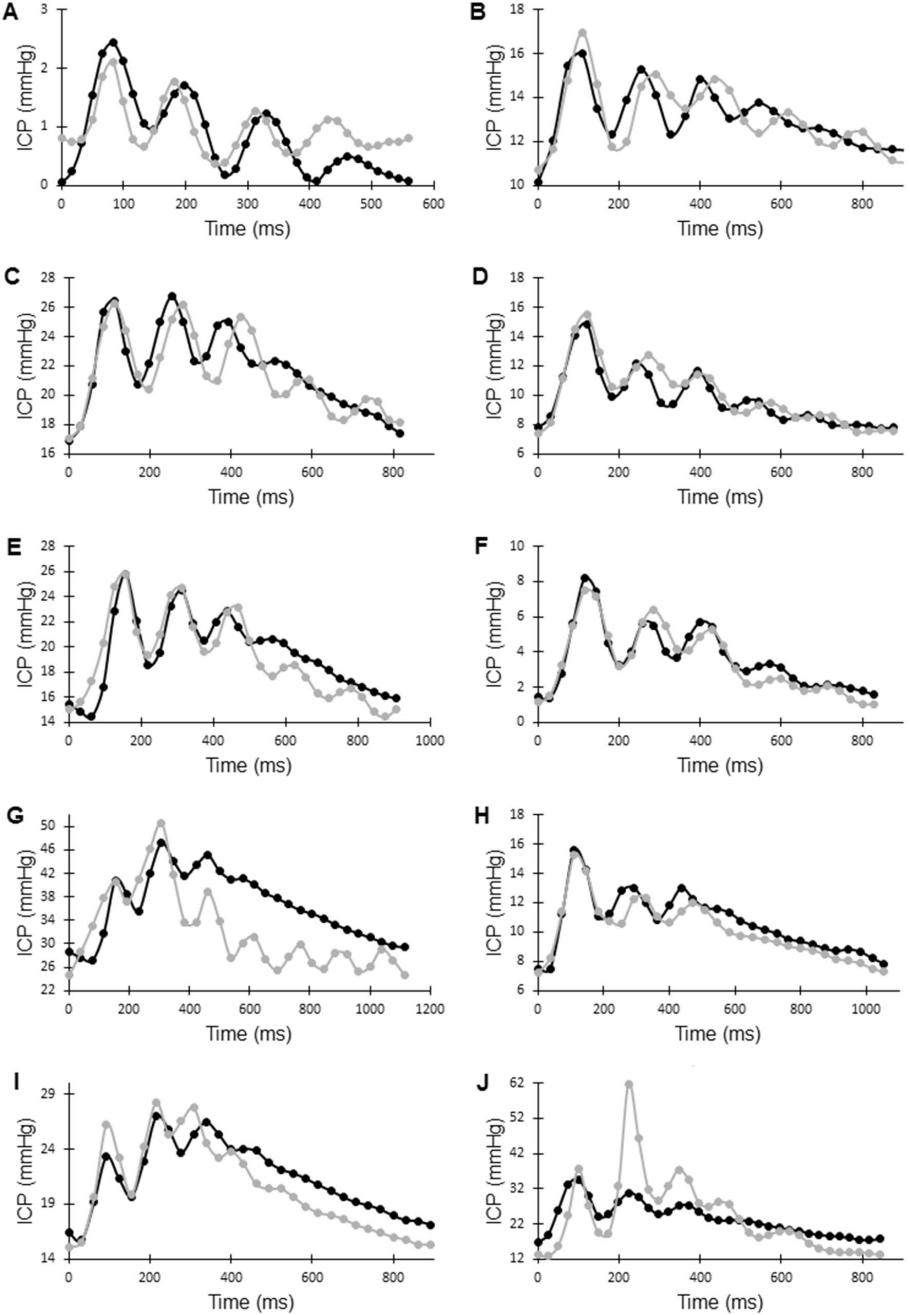
Intracranial pressure curves (ICP) from ten patients (**a**–**j**) with ICP measurements and simultaneous MRI flow measurements. Y axis is the ICP in mmHg. Black represent each single patients ICP curve and the grey curve is the model derived ICP. X-axis is the time of a cardiac cycle. A good fit is achieved in all individuals except J, which has a divergent arterial inflow pattern

**Fig. 3:**
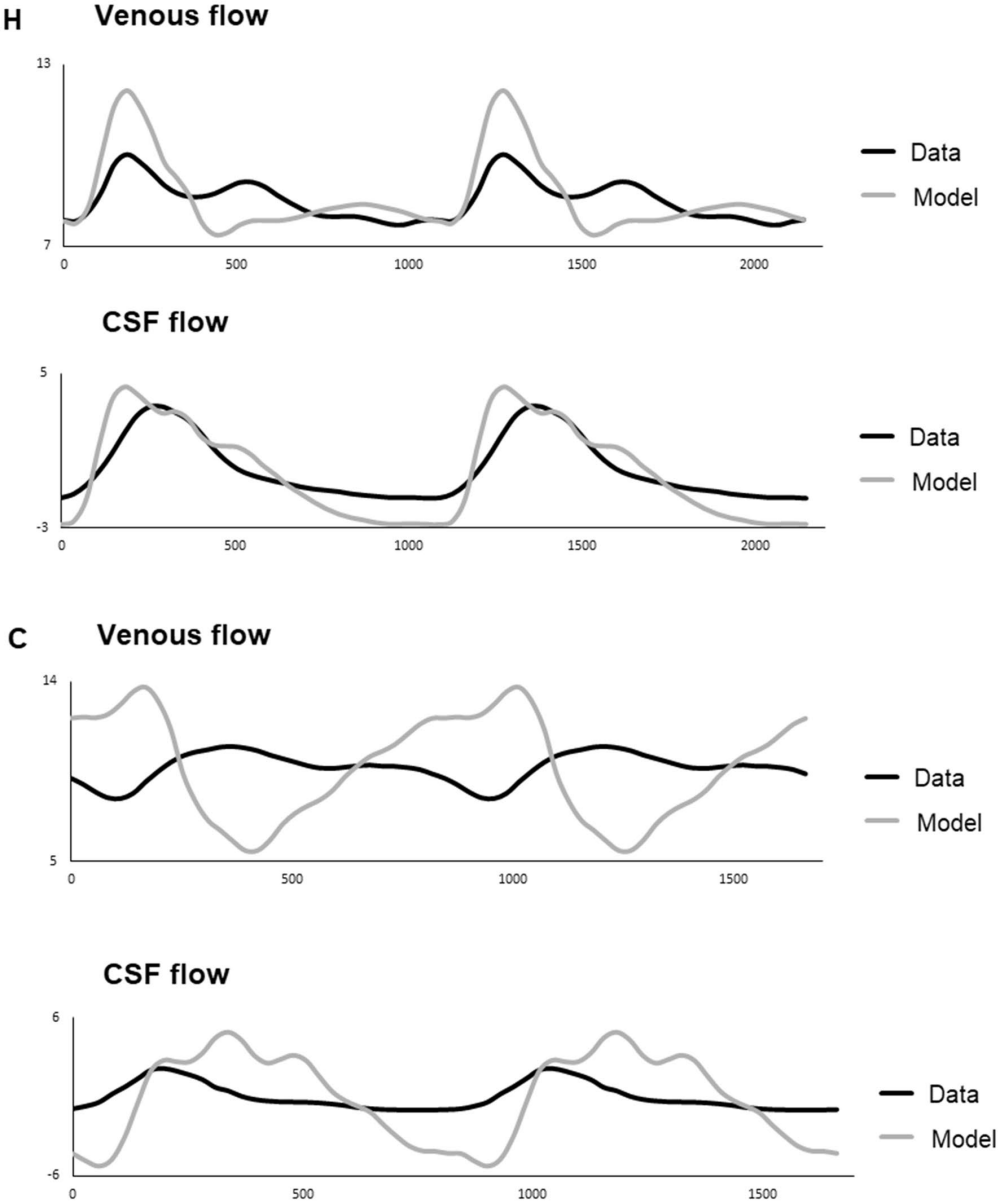
Venous and CSF flow curves from two individuals (H and C). Each patient has two graphs, the upper graph is the flow in the internal jugular veins and the lower is the flow over the foramen magnum. Y axis is the flow in ml/s and the x-axis is in ms showing two cardiac cycles. Black represents the single patients MRI measured flow and grey is data calculated from the mathematical model

10 examinations were eligible for inclusion. nine were males and the mean age was 49 ± 11 years. The mean ICP value was 16 ± 10 mmHg. CBF was 646 ± 199 ml/min. Main diagnosis included six traumatic brain injury (TBI), two subarachnoid haemorrhage, one meningitis and one obstructive hydrocephalus due to a tumour. In all patients it was possible to achieve the multiple peak pattern of the ICP curve as normally observed. Comparisons between measured ICP data and values predicted by the mathematical model are shown in Fig. 2. Examples of output data from the model regarding venous outflow and CSF flow over the foramen magnum as well as measured values are shown in Fig. 3. A high degree of correlation between measured ICP curves and model curves could be achieved, with a mean R2 value of 0.73 ± 0.17. In one individual (J) it was not possible to fit the amplitude, only the frequency of the ICP peaks. Mean ICP curve correlation and output data for each individual are presented in Table 1. There was a temporal displacement between the venous outflow data curve and the modelled venous outflow curve in 4 of the patients. This was observed in lesser extent regarding the CSF outflow curves. Examples of outflow curves are presented in Fig. 3. Comparing the individuals with a P2 > P1 with the individuals with a P1 < P2 there was a significant difference in parameter P_0_, which was higher in individuals with P2 > P1 (p = 0.01). There was no such significant difference in any of the other parameters. Mean parameter values and the set parameter values to achieve the ICP curves are presented in Table 2.

**Table 1:** Results from the mathematical model and measured data. Correlation between measured ICP curve and modeled ICP curve (ICP R^2^) and mean flow values with range

	ICP R^2^	Vein flow data (ml/s)	Vein flow model (ml/s)	CSF flow data (ml/s)	CSF flow model (ml/s)
A	0.53	13.93 (11.79–16.12)	13.59 (8.14–19.00)	0.13 (− 2.34 to 1.71)	0.18 (− 11.35 to 6.37)
B	0.73	4.33 (3.22–5.17)	4.33 (2.54–5.92)	0.08 (− 1.57 to 0.78)	0.09 (− 2.96 to 2.30)
C	0.74	9.70 (8.15–10.75)	9.76 (5.55–13.72)	0.02 (− 2.14 to 1.02)	0.10 (− 4.90 to 5.26)
D	0.90	13.06 (7.66–17.65)	13.09 (11.58–18.90)	0.16 (− 1.21 to 2.36)	0.00 (− 3.11 to 4.24)
E	0.78	10.63 (7.80–12.59)	10.47 (5.84–14.44)	− 0.26 (− 1.09 to 1.79)	− 0.17 (− 5.85 to 5.88)
F	0.91	10.23 (8.53–12.18)	10.10 (6.71–16.30)	0.13 (− 2.06 to 1.39)	0.10 (− 3.41 to 3.33)
G	0.39	9.51 (8.98–10.08)	9.08 (6.16–12.26)	− 0.03 (− 1.04 to 3.16)	0.40 (− 2.61 to 5.61)
H	0.91	8.53 (7.71–10.03)	8.66 (7.39–12.13)	− 0.07 (− 3.28 to 1.46)	0.09 (− 4.29 to 2.82)
I	0.83	10.25 (5.70–15.51)	10.73 (8.77–16.52)	− 0.53 (− 2.75 to 0.49)	0.02 (− 4.29 to 2.82)
J	0.60	17.50 (14.02–23.58)	17.27 (12.54–23.56)	0.06 (− 7.71 to 3.20)	0.20 (− 24.62 to 7.50)

**Table 2:** Mean parameter settings, excluding individual J, and the parameter settings in one individual (H)

Parameter	Mean value	H
*e*_*a0*_	3.33 ± 0.94	3
*R*_*av0*_	12.54 ± 11.78	9.1
*R*_*v0*_	0.54 ± 0.21	0.65
*e*_*c0*_	101.17 ± 80.36	90
*e*_*s*_	3.38 ± 1.09	3.5
*L*_*cs*_	0.10 ± 0.08	0.08
*R*_*cs*_	0.60 ± 0.42	0.45
$\rho_{l}$	6.03 ± 15.55	2
*p*_*0*_	13.66 ± 8.53	9.6
$b_{{L_{s} }}$	0.01 ± 0.02	0.015
$a_{{R_{s} }}$	0.37 ± 0.49	0.6
*Lv*	0.14 ± 0.09	0.14
$\tau_{reg}$	1.31 ± 0.95	1
*k*_*ea*_	0.05 ± 0.05	0.005
$n_{{e_{a} }}$	23 ± 0	23
*m*_*ea*_	1.95 ± 0	1.95
$n_{{R_{av} }}$	36 ± 0	36
$m_{{R_{av} }}$	2 ± 0	2
*e*_*v1*_	1 ± 0	1
*e*_*v2*_	1.48 ± 1.25	1
*m*_*ev1*_	1.89 ± 0.31	2
*m*_*ev2*_	1.87 ± 0.38	2
$m_{{R_{v} }}$	2.89 ± 1.45	2
*k*_*ec*_	1.56 ± 1.40	1

## Discussion

The causes of the ICP curve morphology has been under debate for decades [5]. When the reason for the different ICP waveforms are understood it might be possible to extract clinical important information from the ICP curve. Previous focus has been put on the transfer of the ABP curve intracranial, possibly with dampening, causing the multitude of peaks observed normally. Lately it has been suggested that the morphology could be created by the flow of blood and CSF into and out from the cranial cavity, causing small shifts in intracranial volume [9, 10] and modulated by intracranial pathologies.

To test this hypothesis experimentally in human subjects is, with today’s technology, virtually impossible, though cine phase contrast MRI has opened up for a higher temporal and flow resolution in measurements. Mathematical modelling is used as a method to mimic the reality, thereby making it possible to achieve a deeper understanding of physiological processes circumventing the ethical and practical problems coherent with research in this field. The method has previously been used in this area mainly in order to understand the compliance of the cranial cavity over a longer time period [22–24]. Though models studying the intracranial hydrodynamics over the cardiac cycle have been suggested, these have not been used to analyse the ICP curve morphology [20, 21]. All mathematical models are in some way a simplification of the reality. The model we constructed was built from what is known about physiological properties regarding the intracranial cavity as well as applied physics. The present model resembles by large the one proposed by Ursino [22] but has been further developed in order to examine the response over one cardiac cycle.

An important feature of the described model is the introduction of inertia. This causes a delay in the hydrodynamic response to pressure changes within the intracranial compartments could cause oscillations corresponding to the peaks on the intracranial pressure curve. In previous studies there has constantly been observed a delay between arterial inflow and venous and CSF outflow from the cranial cavity [9, 10], implying this inertia in the cranio-spinal hydrodynamic system. To replicate the peaks of the ICP curve, this inertia can not be omitted from the model.

An interesting finding in our study is that it was possible to mimic the morphology of the measured ICP curve in all patients with each patient’s specific arterial cranial inflow. This implies that the peaks could be the result of the inherent physiological properties of the intracranial cavity, depending on the inflow of arterial blood and the compensatory mechanisms within the intracranial cavity. Despite the simplification of the model it was possible to reproduce the approximate frequencies and amplitudes of the ICP curve form to great accuracy in all subjects with one exception, where only the frequency could be reproduced. This case is discussed later.

Examining the parameters, eight were not adjusted in any individual if individual J is omitted (Table 2) to achieve a model ICP curve resembling the measured ICP curve. It is reasonable to omit individual J when analysing the parameters, since the measurements in this individual probably are erroneous as explained further below. The effects of the different parameters are difficult to predict due to the complexity of the model, but it is clear that P_0_ (pressure outside of the model, affecting the model) has a profound effect on the ICP curve. P_0_ was the only parameter which significantly differed between individuals with a P2 amplitude higher than the P1 amplitude. Parameters e_c0_ (elastance of CSF compartment) and L_cs_ (average impedance to flow from CSF to spinal compartment) seems to differ between the two groups, though not significantly. The effect of P0 probably reflects the parameters impact on compliance. The data must however be interpreted cautiously due to the small amount of observations.

It could be argued that a mathematical model can be constructed to fit any set of values, adjusting it and thereby not correspond to the reality. It is of utmost importance that the model is constructed from known physiological principles, which we do believe we have achieved. The resemblance to Ursinos model [22], based on the same physiological principles, is therefore not a surprise but strengthens the validity of the model. The validity of the model can be examined by comparing the output data with measured values. The output values should correlate to the measured and be within reasonable physiological values. Looking at the graphs plotting measured values of venous and CSF outflow (Fig. 3), these seem to follow the modelled values in six of the cases, such as individual H, and with temporal shifts of the curves in the other cases. The model also seems to calculate a larger range of flow values, though the ranges are within physiological reasonable values. It could not be expected that they would fit perfectly, since the model is a simplification and there are possible errors in the measurements as discussed below. In testing the model, we used the measured ICP curve as a reference, adjusting the parameters to achieve a as high degree of correlation as possible. The output data were treated only as control values. It is possible that if we had tried to fit also the venous outflow and CSF flow data, a better fit regarding these data would have been achieved with a slight impact on the ICP curve. The main purpose of this study was however to explore whether the model could reproduce the ICP curves to a high degree and therefore we choose not to do this.

It is possible that different input parameters than those set in each individual could produce an ICP curve also in close resemblance of the measured ICP curve. The output variables could therefor differ depending on chosen input variables despite resulting in similar ICP curves. In this study we explored if it was at all possible to mimic the ICP curve morphology through mathematical modelling of the intracranial physiology. The resulting differences between measured and model predicted output and the effects on the ICP curve depending on the set parameters should be further explored in future studies.

In one patient (J) it wasn’t possible to make a fit regarding the amplitude of the ICP curve, the frequency of the peaks could be reproduced. This individual has a divergent arterial cerebral inflow pattern with a second flow peak, secondary to the dicrotic notch, higher than the first. We have not previously come across a patient with this kind of CBF pattern. One reason could be an error during the MRI measurement, but we have not been able to sufficiently identify this error to exclude this patient.

Regarding the outflow patterns of CSF and venous outflow they follow the predicted values in close adjunct. Predicted values are all close to the range of measured values, except in individual J and peaks in flow are close to measured peaks in most of the examinations. The small variation in time and flow observed between measured values and predicted values could be due to inaccuracies caused by over simplifications in the mathematical model, but they could also be the result of errors in the measurements. All changes of flow and ICP are measured over very small time increments and the exact starting point, t = 0, of the ICP and flow measurements might be slightly different between the measurements, which could introduce errors. Indeed, the small net flows of CSF over the cardiac cycle that can be observed is probably due to errors in measurements.

Venous outflow was measured in the jugular veins, while the model actually predicts flow at the point where the venous blood leaves the cranial cavity. The venous flow at this point could differ from the measured flow [17].

## Conclusion

We present a mathematical model based on known physiological principles which is able to mimic the physiological properties of the intracranial cavity regarding ICP, arterial blood inflow, venous blood outflow and CSF flow in and out from the cranial cavity.

Using this model, it is possible to mimic the ICP curve morphology of the individual and this strengthens the hypothesis that the ICP curve form is caused by the arterial blood inflow and the craniospinal systems physiological and/or pathophysiological properties.

## Figures and Tables

**Fig. 4:**
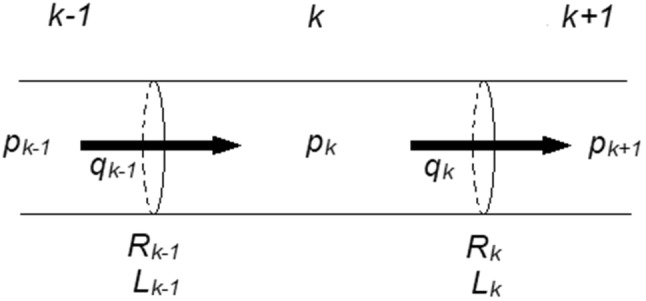
A compartmental model. An illustration of flow through a compartmental model. Compartments are identified as k, k-1 and k + 1. Pressure (p), flow (q), resistance (R) and impedance (L) are all denoted by their compartment

**Fig. 5:**
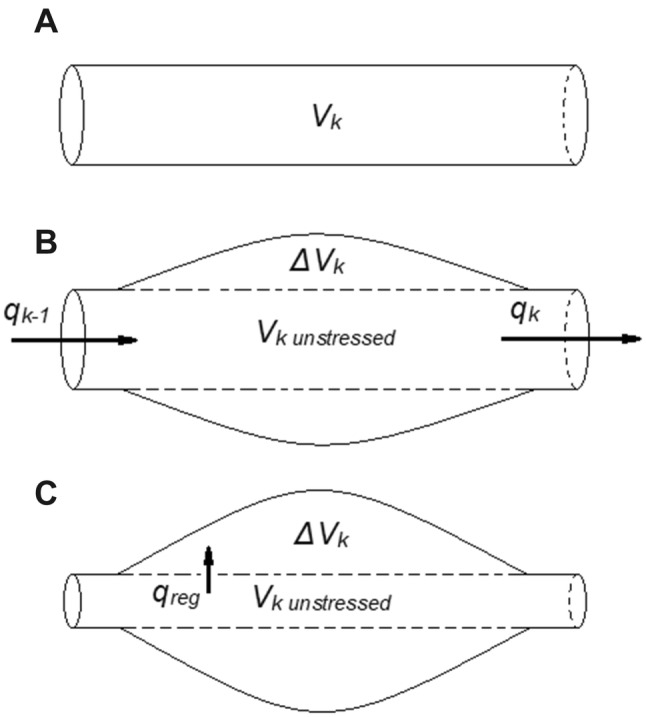
Illustration of stressed volume. In (**a)** a vessel is unstressed, the volume in that compartment is V_k_ and the internal pressure p_k_ equals the external pressure, p_outside_. In (**b)** a larger inflow q_k-1_ than outflow q_k_ causes a volume expansion and a pressure increase in the vessel, which expands the stressed volume (ΔV_k_) due to the increased transmural pressure, p_k_−p_outside_. V_k_ now equals ΔV_k_ + V_k_, _unstressed_. (**c)** illustrates the effect of an additional local regulation, which simultaneously adjust the arterial compliance c_k_ and changes the ratio of ΔV_k_ and ΔV_k_, _unstressed_. This redistribution of fluid is described by a flow q_reg_ between the unstressed and the stressed volumes. We emphasize that the flow q_reg_ also dependents on the compliance of the specific compartment, which is the inverse elastance of the vessel, e_k_ = 1/c_k_

**Fig. 6:**
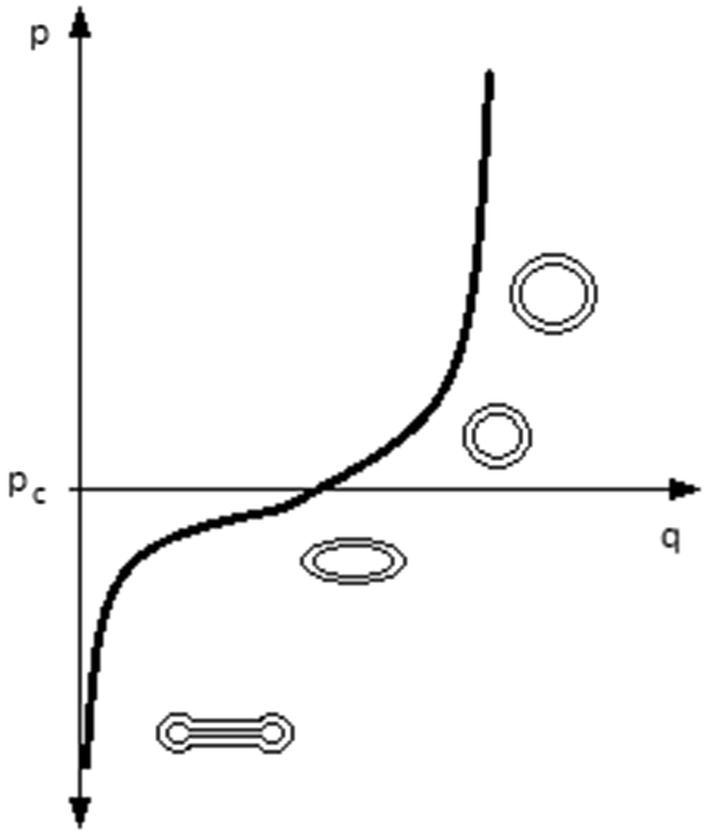
The typical venous pressure-flow relation. Flow (q) through a vein as a function of venous pressure (p). At high pressures the vein is distended and circular. At the pressure P_c_ the vein starts to collapse, becoming increasingly oval in shape, until almost totally compressed. After Rideout, 1991

## Appendix

Blood flow through distensible vessels may be described by a number of subsequent compartmental equations. (Figure 4) The equation:

$$L_{k} \frac{{dq_{k} (t)}}{dt} = p_{k} (t) - p_{k + 1} (t) - R_{k} q_{k} (t)$$

describes the flow *q*_*k*_ from compartment *k* to compartment *k *+ *1* where *L*_*k*_ is the inertia (impedance) and *R*_*k*_ the resistance to this flow in the compartment *k*.

The second equation is a conservation equation. It describes the volume in compartment *k* as a function of the flow to and from this compartment.

$$\frac{{dV_{k} (t)}}{dt} = q_{k - 1} (t) - q_{k} (t)$$

This equation states that the time derivate of volume in compartment *k* equals the difference of the time dependent flow into and out from compartment *k*.

The third equation relates the transmural pressure of the compartment to the stressed volume of the compartment. Transmural pressure is here defined as *p*_*k*_*−p*_*outside*_. The stressed volume (Δ*V*_*k*_) is the volume in the compartment that is the consequence of a pressure within the compartment higher than that of the surrounding pressure, Δ*V*_*k*_ = *V*_*k*_−*V*_*k unstressed*_. The importance of calculating the stressed volume lies in the fact that it is this volume that interacts with the surroundings regarding pressure. (Figure 5).

The relationship is usually described by the equation

$$\Delta V_{k} = c_{k} \left( {p_{k} (t) - p_{outside} } \right),$$

where compliance of the compartment (*C*_*k*_) is introduced, but frequently the non-linear relation

$$\Delta V_{k} = \log \left( {c_{k} \left( {p_{k} (t) - p_{outside} } \right)} \right),$$

is used [24].

Compliance hence describes the relationship between volume and pressure within the compartment. The inverse compliance of the compartment is termed the elastance (*e*_*k*_), whereas the inverse resistance of the compartment is conductivity (*ρ*_*k*_). In general, all these parameters depend on material properties, universal constants and radii, thus volume of the tube element considered. In case of Poiseuille flow such relations may be derived analytically (*L*_*k*_~ *V*_*k*_^−1^, *R*_*k*_~ *V*_*k*_^−2^ and *c*_*k*_~ *V*_*k*_^2/3^) but in general this is not possible [35].

### The model

A model simulating the intracranial hydrodynamics was created using four compartments, the arterial compartment, the venous compartment, the CSF compartment and the spinal compartment.

Applying the general equations above on the proposed model we arrive at a system of eight specific differential equations, which on closed form read,

1 $$\Delta V^{\prime}_{a} = q_{a} - q_{av} + q_{reg}$$

2 $$\Delta V^{\prime}_{v} = q_{av} - q_{v}$$

3 $$\Delta V^{\prime}_{c} = q_{a} - q_{v} - q_{s}$$

4 $$\Delta V^{\prime}_{s} = q_{s} - q_{l}$$

5 $$L_{s} q^{\prime}_{s} = e_{c} \Delta V_{c} - e_{s} \Delta V_{s} - R_{s} q_{s}$$

6 $$L_{v} q^{\prime}_{v} = e_{v} \Delta V_{v} + e_{c} \Delta V_{c} - R_{v} q_{v}$$

7 $$q^{\prime}_{am} = q_{reg}$$

8 $$V^{\prime}_{a0} = - q_{reg}$$

where

9 $$q_{av} = \rho_{av} \left( {e_{a} \Delta V_{a} - e_{v} \Delta V_{v} } \right)$$

10 $$q_{l} = \rho_{l} e_{s} \Delta V_{s}$$

and

11 $$q_{reg} = \frac{{q_{a} - q_{am} }}{{\tau_{reg} }}$$

Equations (1)–(4) describe the stressed volume changes over time in the four compartments, arterial (*a*), venous (*v*), CSF (*c*) and spinal (*s*), depending on the flow between these compartments.

*q*_*reg*_, as introduced in Eq. (1), is the flow from unstressed to stressed volume within the arterial compartment. It is calculated through Eq. (6), further described below. In Eq. (4) the model includes a possible leak from the spinal canal out from the model. This leak is however insignificant on the time scale of one heartbeat.

In Eq. (5) impedance is introduced to the flow of CSF over the foramen magnum. The flow is driven by the pressure difference between the CSF compartment and the spinal compartment and opposed by the resistance to this flow. Both may depend on the direction of flow (see below).

Equation (6) introduces impedance to the flow from the venous compartment out from the model, i.e. to the systemic venous circulation. This flow is dependent on the pressure in the venous compartment, which indirectly includes the pressure put on this compartment by the pressure in the CSF compartment and the resistance to this flow.

Equation (7) defines the mean arterial flow *q*_*am*_ as a weighted mean over time using an exponential weight kernel function with time constant *τ*_*reg*_. Equivalently, the derivative of *q*_*am*_ becomes *q*_*reg*_ defined in (9). Thus, *q*_*reg*_ dependents on previous flow, providing the model with a function to adapt to rapid changes in flow through myogenic response. The concept is further developed below.

Equation (8) stipulates that the derivative of the unstressed volume, i.e. the change in unstressed volume (*V*_*a0*_*′)* dependents on the flow from the unstressed to the stressed volume within the arterial compartment. This equation conserves the arterial fluid in each time step of the integration when solving the equations numerically.

Equation (9) describes the flow from the arterial to the venous compartment as dependent on the conductance of this flow and the difference in pressure between the two compartments.

Equation (10), in the same way, defines the leak from the spinal compartment out from the model as dependent on the conductance of this flow and the pressure in the spinal compartment.

Equation (11) defines *q*_*reg*_ as dependent on the weighted mean arterial flow and the current arterial flow. The mean arterial flow is weighted with a decaying history, the latter having a time constant *τ*_*reg*_. Combining this equation with Eq. (1) implies a myogenic control, where the stressed volume is dependent on the arterial compartment’s muscular contraction, which is dependent on previous inflow.

We emphasize that the parameters are not all treated as being constant and in fact we will allow some of these to depend on the state variables (the stressed volumes), since these are known to vary with vessel radii [35].

#### Pressures

Equations (12)–(15) state that stressed volumes and transmural pressures are proportional, with proportionality constant given by the vessel elastances in analogy with Ohm’s law. However, later on these elastance constants are allowed to vary with the unstressed volumes [35]. After the previous equations are solved the pressure in each compartment may be calculated through these.

12 $$p_{a} = e_{a} \Delta V_{a} + p_{c}$$

13 $$p_{v} = e_{v} \Delta V_{v} + p_{c}$$

14 $$p_{c} = e_{c} \Delta V_{c} + p_{0}$$

15 $$p_{s} = e_{s} \Delta V_{s} + p_{0}$$

#### Varying parameters

As stated above, some of the parameters in the flow and volume equations will depend on the state of the model. The varying parameters (Table 3) are calculated through Eqs. (16)–(23).

**Table 3:** The parameters that are used to change the properties of the mathematical model

Parameter	Explanation
*e*_*a0*_	Elastance arterial compartment
*R*_*av0*_	Resistance to flow from arterial to venous compartment
*R*_*v0*_	Resistance to outflow from the venous compartment
*e*_*c0*_	Elastance CSF compartment
*e*_*s*_	Elastance spinal compartment
*L*_*cs*_	Average impedance to flow from CSF to spinal compartment
*R*_*cs*_	Average resistance to flow from CSF to spinal compartment
$\rho_{l}$	Conductance of leak flow from spinal compartment
*p*_*0*_	Pressure outside of the model
$b_{{L_{s} }}$	Asymmetry L_cs_-factor describing the L_cs_ impedance dependence on flow direction
$a_{{R_{s} }}$	Asymmetry Rcs-factor describing the Rcs resistance dependence on flow direction
*L*_*v*_	Impedance to venous outflow from the model
$\tau_{reg}$	Time constant describing the weights in the weighted average flow q_am_
*k*_*ea*_	Constant describing how the elastance artery depends on q_reg_
$n_{{e_{a} }}$	ΔV_a_ normalizing constant in describing how artery elastance depends on ΔV_a_
*m*_*ea*_	Power describing how artery elastance depends on ΔV_a_
*nR*_*av*_	ΔV_a_ normalizing constant in describing how artery resistance depends on ΔV_a_
*mR*_*av*_	Power describing how artery resistance depends on ΔV_a_
*e*_*v1*_	Coefficient 1 of venous elastance describing compression
*e*_*v2*_	Coefficient 2 of venous elastance describing dilatation
$m_{{e_{v1} }}$	Power describing how e_v_ depends on ΔV_v_ during compressions
$m_{{e_{v2} }}$	Power describing how ev depends on ΔVv during dilations
$m_{{R_{v} }}$	Power describing how venous outflow resistance depends on ΔVv
*k*_*ec*_	Coefficient of elastance CSF, describing how ec depends on ΔVc

16 $$q_{reg} = \frac{{\left( {q_{a} - q_{am} } \right)}}{{\tau_{reg} }},\,\,\,for\,V_{a0} > 0$$

otherwise,

$$q_{reg} = 0$$

17 $$e_{a} = e_{a0} \left( {\frac{{n_{{e_{a} }} }}{{\Delta V_{a} }}} \right)^{{m_{ea} }} \exp ( - k_{ea} q_{reg} )$$

18 $$R_{av} = R_{av0} \left( {\frac{{n_{{R_{av} }} }}{{V_{a0} + \Delta V_{a} }}} \right)^{{m_{{R_{av} }} }}$$

19 $$R_{v} = {{R_{v0} } \mathord{\left/ {\vphantom {{R_{v0} } {\Delta V_{v}^{{m_{{R_{v} }} }} }}} \right. \kern-0pt} {\Delta V_{v}^{{m_{{R_{v} }} }} }}$$

20 $$e_{v} = - \frac{{e_{v1} \left( {\Delta V_{v} } \right)^{{m_{{e_{v1} }} }} }}{{V_{v0} + \Delta V_{v} }},\,\,\,{\text{for}}\,\Delta V_{v} < 0$$

$$e_{v} = e_{v2} \left( {\Delta V_{v} } \right)^{{m_{{e_{v2} }} }} ,\,\,\,{\text{for}}\,\Delta V_{v} \ge 0$$

21 $$e_{c} = e_{c0} \exp \left( {k_{ec} \Delta V_{c} } \right)$$

22 $$R_{s} = R_{{cs}} \left( {1 + a_{{R_{s} }} } \right),\,{\text{for}}\,q_{s} > 0$$

$$R_{s} = R_{cs} \left( {1 - a_{{R_{s} }} } \right),\,\,\,{\text{for}}\,q_{s} \le 0$$

23 $$L_{s} = L_{cs} \left( {1 - b_{{L_{s} }} } \right),\,\,\,{\text{for}}\,q_{s} > 0$$

$$L_{s} = L_{cs} \left( {1 + b_{{L_{s} }} } \right),\,\,\,{\text{for}}\,q_{s} \le 0$$

In Eq. (16) we state that *q*_*reg*_ should be calculated according to Eq. (11) as long as the unstressed volume in the arterial compartment is positive. By setting this condition, we define the flow to the stressed volume as dependent on *q*_*reg*_ only if there is any fluid to flow.

Equation (17) states that the elastance of the arterial compartment is dependent on the elastance in the unstressed state (*e*_*a0*_) and the power of the stressed volume and the elastance due to muscular resistance to distension in the arterial wall (*m*_*ea*_). It is also exponentially related to the *q*_*reg*_ and decaying, described by a negative coefficient (*k*_*ea*_). In sum, this equation tells us that the elastance at any given moment is dependent on the stressed volume and the flow from unstressed to stressed volume at that moment.

In Eq. (18) we state that the resistance to flow from the arterial to the venous compartment varies depending on a constant (*k*_*va*_), total volume in the arterial compartment and a myogenic resistance factor (*m*_*Rav*_). The result is that an increase in arterial radius due to increased arterial pressure will decrease resistance in the compartment. This follows Poiseuille’s law.

Equation (19) states the same regarding resistance to venous outflow from the model. Resistance is dependent on the stressed volume within the compartment and a resistance factor (*m*_*Rv*_).

Equation (20) determines the elastance in the venous compartment. The veins are thin walled, with low transmural pressures and are susceptible to the intracranial pressure. Therefore, these equations follow a nonlinear relationship, based on empirical knowledge of this pressure–volume relation, which is related to the theory of flow in collapsible tubes [28–32, 35]. The vessels change shape depending on the degree of collapse This affects resistance to flow and thereby flow through the vessels. The pressure-flow relation follows different curves, depending on whether it is collapsed or dilated. Since the change in venous flow is dependent on venous elastance (Eq. (6)) this effect is modelled by making elastance dependent on the degree of venous collapse or dilation, respectively.

The curve is modelled using two different equations, depending on the venous stressed volume. A set of constants then determine the slope of the two curves (*e*_*v1*_, *e*_*v2*_, *m*_*ev1*_ and *m*_*ev2*_) as illustrated in Fig. 6.

Equation (21) calculates the elastance of the CSF compartment depending on the elastance coefficient and the stressed volume within the CSF compartment, using an exponential elastance function in the stressed volume Δ*V*_*c*_.

Equations (22) and (23) concern the resistance and the impedance to the flow from the CSF compartment to the spinal compartment. By making them dependent on the direction of flow (the sign of *q*_*s*_) it is possible to set different resistance and impedance depending on the direction of the flow.

## References

[1] Guillaume J, Janny P (1951). Continuous intracranial manometry; physiopathologic and clinical significance of the method. Presse Med.

[2] Lundberg N (1960). Continuous recording and control of ventricular fluid pressure in neurosurgical practice. Acta Psychiatr Scand Suppl.

[3] Carney N, Totten AM, O’Reilly C, Ullman J, Hawryluk G, Bell M (2017). Guidelines for the management of severe traumatic brain injury. Neurosurgery.

[4] Sorrentino E, Diedler J, Kasprowicz M, Budohoski KP, Haubrich C, Smielewski P, Outtrim JG, Manktelow A, Hutchinson PJ, Pickard JD, Menon DK, Czosnyka M (2012). Critical thresholds for cerebrovascular reactivity after traumatic brain injury. Neurocrit Care.

[5] Balédent O, Czosnyka M, Czosnyka Z (2018). Brain pulsations elightened. Acta Neurochir.

[6] Hamit H, Beall A, DeBakey M (1965). Hemodynamic influences upon brain and cerebrospinal fluid pulsations and pressures. J Trauma.

[7] Zou R, Park E, Kelly E, Egnor M, Wagshul M, Madsen J (2008). Intracranial pressure waves: characterization of a pulsation absorber with notch filter properties using systems analysis: laboratory investigation. J Neurosurg Pediatr.

[8] Wagshul M, Kelly E, Yu B, Garlick T, Zimmerman M, Egnor M (2009). Resonant and notch behaviour in intracranial pressure dynamics. J Neurosurg Pediatr.

[9] Alperin NJ, Lee SH, Loth F, Raksin PB, Lichtor T (2000). MR-Intracranial pressure (ICP) a method to measure intracranial elastance and pressure noninvasively by means of MR imaging: baboon and human study. Radiology.

[10] Unnerbäck M, Ottesen JT, Reinstrup P (2018). ICP curve morphology and intracranial flow-volume changes: a simultaneous ICP and cine phase contrast MRI study in humans. Acta Neurochir.

[11] Hu X, Glenn T, Scalzo F, Bergsneider M, Sarkiss C, Martin N, Vespa P (2010). Intracranial pressure pulse morphological features improved detection of decreased cerebral blood flow. Physiol Meas.

[12] Unnerbäck M, Bloomfield EL, Söderström S, Reinstrup P (2018). The intracranial pressure curve correlates to the pulsatile component of cerebral blood flow. J Clin Monit Comput.

[13] Bryant DJ, Payne JA, Firmin DN, Longmore DB (1984). Measurement of flow with NMR imaging using a gradient pulse and phase difference technique. J Comput Assist Tomogr.

[14] Marks MP, Pelc NJ, Ross MR, Enzmann DR (1992). Determination of cerebral blood flow with a phase-contrast cine MR imaging technique: evaluation of normal subjects and patients with arteriovenous malformations. Radiology.

[15] Balédent O, Henry-Feugeas M, Idy-Peretti I (2001). Cerebrospinal fluid dynamics and relation with blood flow: a magnetic resonance study with semiautomated cerebrospinal fluid segmentation. Invest Radiol.

[16] Alperin N, Lee SH, Sivaramakrishnan A, Hushek SG (2005). Quantifying the effect of posture on intracranial physiology in humans by MRI flow studies. J Magn Reson Imaging.

[17] Stoquart-ElSankari S, Lehmann P, Vilette A, Czosnyka M, Meyer M, Deramond H, Balédent O (2009). A phase-contrast MRI study of physiological cerebral venous flow. J Cereb Blood Flow Metab.

[18] Martin B, Reymond P, Novy J, Baledent O, Stergiopulos N (2012). A coupled hemodynamic model of the cardiovascular and cerebrospinal fluid system. Am J Physiol Heart Circ Physiol.

[19] Toro E, Muller L, Cristini M, Menegatti E, Zamboni P (2015). Impact of jugular vein valve function on cerebral venous hemodynamics. Curr Neurovasc Res.

[20] Linninger A, Tsakiris C, Zhu D, Xenos M, Roycewicz P, Danziger Z, Penn R (2005). Pulsatile cerebrospinal fluid dynamics in the human brain. IEEE Trans Biomed Eng.

[21] Ambarki K, Baledent O, Kongolo G, Bouzerar R, Fall S, Mayer M (2007). A new lumped-parameter model of cerebrospinal hydrodynamics during the cardiac cycle in healthy voulenteers. IEE Trans Biomed Eng.

[22] Ursino M, Lodi C (1997). A simple mathematical model of the interaction between intracranial pressure and cerebral hemodynamics. J Appl Physiol.

[23] Czosnyka M, Piechnik S, Richards H, Kirkpatrick P, Smielewski P, Pickard J (1997). Contribution of mathematical modelling to the interpretation of bedside tests of cerebrovascular autoregulation. J Neurol Neurosurg Psychiatry.

[24] Marmarou A, Shulman K, Rosende RM (1978). A nonlinear analysis of the cerebrospinal fluid system and intracranial pressure dynamics. J Neurosurg.

[25] Greitz D, Wirenstam R, Franck A, Nordell B, Thomsen C, Ståhlberg F (1992). Pulsatile brain movement and associated hydrodynamics studied by magnetic resonance phase imaging. Neuroradiology.

[26] Laganà M, Shepherd S, Cecconi P, Beggs C (2017). Intracranial volumetric changes govern cerebrospinal fluid flow in the Aqueduct of Sylvius in healthy adults. Biomed Signal Process Control.

[27] Granger RA (1995). Fluid mechanics.

[28] Low HT, Chew YT (1991). Pressure/flow relationships in collapsible tubes: effects of upstream pressure fluctuations. Med Biol Eng Comput.

[29] Pedley TJ, Luo XY (1998). Modelling flow and oscillations in collapsible tubes. Theoret Comput Fluid Dynamics.

[30] Pedley TJ, Pihler-Puzovic D (2015). Flow and oscillations in collapsible tubes: physiological applications and low-dimensional models. Sadhana.

[31] Conrad WA (1969). Pressure-flow relationships in collapsible tubes. IEEE Trans Biomed Eng.

[32] Noordegraaf A (1978). Circulatory system dynamics.

[33] Heiberg E, Sjögren J, Ugander M, Carlsson M, Engblom H, Arheden H (2010). Design and validation of segment—a freely available software for cardiovascular image analysis. BMC Med Imaging.

[34] Doepp F, Schreiber SJ, von Münster T, Rademacher J, Klingebiel R, Valdueza J (2004). How does the blood leave the brain? A systematic ultrasound analysis of cerebral venous drainage patterns. Neuroradiology.

[35] Rideout VC (1991). Mathematical and computer modeling of physiological systems.

